# MoveLab^®^: Validation and Development of Novel Cross-Platform Gait and Mobility Assessments Using Gold Standard Motion Capture and Clinical Standard Assessment

**DOI:** 10.3390/s25185706

**Published:** 2025-09-12

**Authors:** Katie Powell, Ahmad Amer, Zornitza Glavcheva-Laleva, Jenny Williams, Caomhnad O’Flaherty Farrell, Finchley Harwood, Peter Bishop, Catherine Holt

**Affiliations:** 1 School of Engineering, Cardiff University, Cardiff CF10 3AT, UKwilliamsj154@cardiff.ac.uk (J.W.);; 2 Agile Kinetic, Newport NP20 1GL, UKpeter.b@agilekinetic.com (P.B.)

**Keywords:** gait, spatiotemporal gait parameters (STPs), STS, TUG, rehabilitation, frailty, validation, wearable, MoveLab^®^

## Abstract

Wearable health assessment devices enable real-time clinical- and home-based patient monitoring. Human gait analysis is a widely accepted musculoskeletal assessment. The 30 s Sit-to-Stand (STS) and Timed-Up-and-Go (TUG) are clinical frailty assessments used alongside gait analysis. This study assessed the reliability and validity of the MoveLab^®^ (Agile Kinetic 2024) approach to measure gait spatiotemporal parameters (STPs), STS, and TUG using a waist-worn mobile phone, compared to the Gold Standard 3D marker-based motion capture (Qualisys AB, Sweden) and the Clinical Standard assessment of the STS and TUG test methods. Movement data, recorded simultaneously for 25 healthy volunteers (14 female and 11 male, Age = 31.8 ± 11.6 yrs) in a Biomechanics Laboratory using the Gold Standard system, the Clinical Standard assessments, and MoveLab^®^, was analyzed using Intraclass Correlation (ICC) and Bland–Altman plots (Python) to quantify the correlations, consistency, and significance across the output parameters. Comparing the methods, the STP consistency ranged from acceptable to good for all the tested parameters (ICC 0.299–0.894). The highest and lowest correlations were cycle time and terminal double support time, respectively. The TUG showed good agreement (ICC 0.757). Generally, an equal number of MoveLab^®^ STS repetitions were observed. MoveLab^®^ demonstrated validity and reliability for a range of key movement parameters using a pouch-worn mobile phone device in healthy adults in a controlled laboratory environment.

## 1. Introduction

The increasing adoption of digital healthcare solutions is reshaping modern medicine, particularly in precision medicine, which tailors the treatment and disease management based on individual variability in genes, environment, and lifestyle. Advances in health data analytics, artificial intelligence (AI), and wearable health devices have driven this transformation, enabling real-time patient monitoring and early disease detection [1]. Cloud-based solutions, combined with machine learning models, are further improving patient care by streamlining data interoperability and clinical workflows, ultimately enhancing accessibility and reducing healthcare costs [2]. As the healthcare industry moves toward remote patient monitoring and AI-driven diagnostics, the demand for scalable, cost-effective, and accessible digital health tools continues to rise.

Digital gait analysis has emerged as a powerful tool in biomechanics, rehabilitation, and sports medicine, enabling more objective assessments beyond the traditional observational methods. Wearable devices such as inertial measurement units (IMUs) and force-sensitive footwear have improved accessibility but still face barriers in terms of cost, data reliability, and usability in real-world settings [3]. AI-enhanced gait analysis, such as machine-learning-based event detection, has demonstrated potential benefits, particularly in patients with neurological impairments such as Parkinson’s disease [4]. Open-source libraries such as Scikit Digital Health offer accessible tools for gait, activity, and sleep data [5], though their implementation often requires technical expertise, which may limit their widespread clinical adoption. Furthermore, the existing IMU-based systems require proper sensor placement and calibration [6], making them less feasible for unsupervised, large-scale use. Among the commercial solutions, smartphone IMU-based movement assessments such as OneStep (Celloscope Inc., New York) have been introduced, which offer highly portable, feasible, and easy-to-use methods based on valid, reliable, and sensitive spatiotemporal gait parameters [7].

Vision-based solutions, such as monocular pose estimation, provide contextual data by mapping full-body movement using an avatar representation. However, these methods introduce their own set of challenges. An accurate pose estimation often requires a controlled environment with good lighting and minimal background clutter [8]. Factors such as loose clothing, which obscures key anatomical landmarks, can also impact the tracking accuracy [9]. Additionally, vision-based systems require sufficient space to keep the subject’s full body within the camera’s field of view, limiting their usability in confined settings [8]. These constraints underscore the trade-offs between sensor-based and vision-based gait analysis technologies.

The traditional methods for measuring human gait patterns, such as optical motion capture (MoCap) systems, force plates, and pressure-sensitive walkways, remain the Gold Standard for gait analysis due to their high precision [10,11,12,13,14]. However, “the expensive equipment and technical expertise necessary to operate a gait laboratory are inaccessible to most clinicians” [9]. As a result, clinical gait assessments often rely on observational methods.

This preliminary study aims to validate the MoveLab^®^ (Agile Kinetic Ltd., Newport UK, 2024) sensor-based gait spatiotemporal parameter (STP) analysis capabilities against a Gold Standard optical MoCap system (Qualisys AB, Gothenburg, Sweden), building on prior validation for vision-based assessments [15]. In addition, this study evaluates the accuracy and reliability of MoveLab^®^ Timed-Up-and-Go (TUG) and Thirty-Second Sit-to-Stand (STS) assessments, compared to the Clinical Standard assessment methods, which are widely recognized indicators of balance, functional mobility, and lower limb strength [16,17,18]. The findings of this study will contribute to the ongoing development of scalable, clinically validated digital mobility assessment tools, with potential applications in clinical, laboratory, and remote healthcare environments.

## 2. Materials and Methods

Twenty-five healthy volunteers (14 Female and 11 Male, Age = 31.8 ± 11.6 yrs) with no history of gait impairment participated in this validation study. Following ethical approval from the relevant Research Ethics Committee (REC), all participants were given an Information Sheet and gave their written Informed Consent on the day of data collection. Participants were recruited via word of mouth within Cardiff University and from the general public. Participants were offered a £25 shopping voucher as compensation for their time. This study was performed in the Musculoskeletal Biomechanics Research Facility (MSKBRF), School of Engineering, Cardiff University, with all data collected in the Clinical Laboratory. Data was collected for all 25 participants over eight weeks and the data collection protocol was the same for each participant, collected in a single session.

The MSKBRF Motion Capture (MoCap) Laboratory equipment comprised twelve Oqus 700+ infrared cameras and two Oqus 210c video cameras (Qualisys AB, Gothenburg, Sweden) for 2D and 3D data capture at 100 Hz and 24 Hz, respectively. The cameras were synchronised using the QTM trigger module via a transistor–transistor logic (TTL) pulse to start and stop recording, with an instrumented walkway comprising six staggered ground reaction force plates (Bertec Inc., Columbus, OH, USA), capturing at 1000 Hz. A standard marker set was implemented and all resulting MoCap data was tracked (Qualisys Track Manager, QTM version 2018.1) and processed through an established, standard 3D Inverse Dynamic Model pipeline (Visual 3D, HAS Motion, ON, Canada).

**Figure 1:**
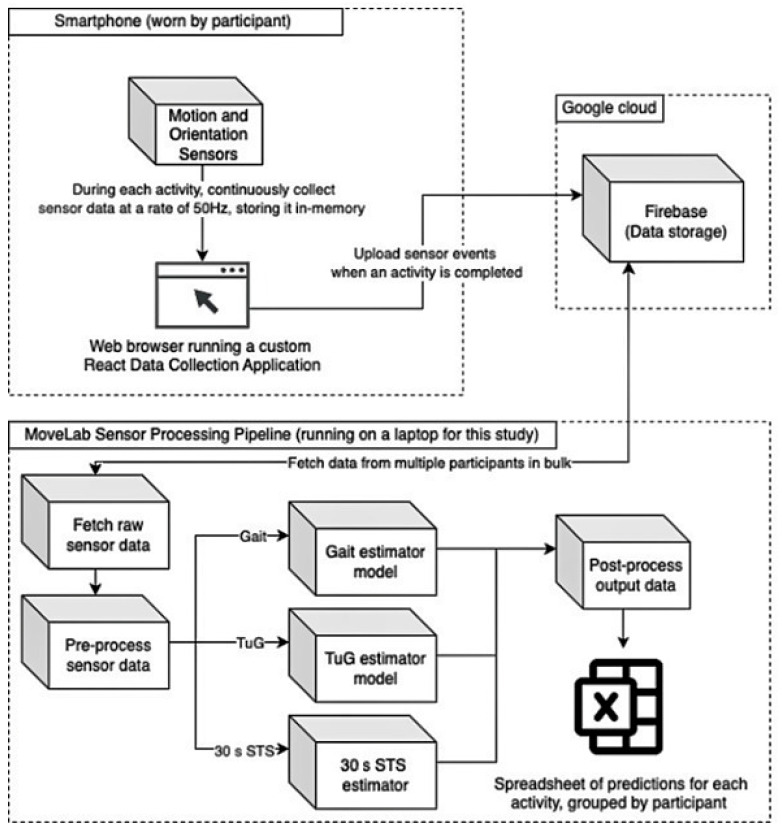
A diagram showing the flow of raw sensor data through the testing process.

Agile Kinetic developed a browser-based React (https://react.dev/ (accessed on 1 September 2023)) Data Collection Application (DCA) for the study, using the smartphone’s built-in inertial sensors to measure acceleration and orientation in three dimensions at a sampling rate of at least 50 Hz. The application enforced a consistent data labelling convention, allowing activities to be paired with the data recorded by the MoCap system. As shown in Figure 1, the raw sensor data from each experimental repetition was stored in the device memory and then uploaded to a secure Firebase database (https://firebase.google.com (accessed on 1 September 2023)). A separate Python (Python Software Foundation. Python Language Reference, version 3.10. Available at https://www.python.org (accessed on 1 September 2023)) processing pipeline, running on a laptop, was used to fetch and process the raw sensor data. This pipeline consisted of four stages: data fetch, pre-processing, processing (which was different for each of the three activity types), and post-processing. The processing pipeline produced a .csv file containing predictions for each activity, grouped by participant for subsequent comparison with the MoCap system outputs.

Each participant was assigned a unique identification number (UID). Participant confidentiality was ensured by using the UID instead of the participant’s name when tagging data in the DCA and QTM.

Participants were asked to wear shorts and a loose vest or t-shirt and to perform all activities barefoot as per the generally accepted protocol for clinical gait analysis data collection [19].

Age and anthropometric information, including height, weight, and left leg length, was recorded for each participant.

**Figure 2:**
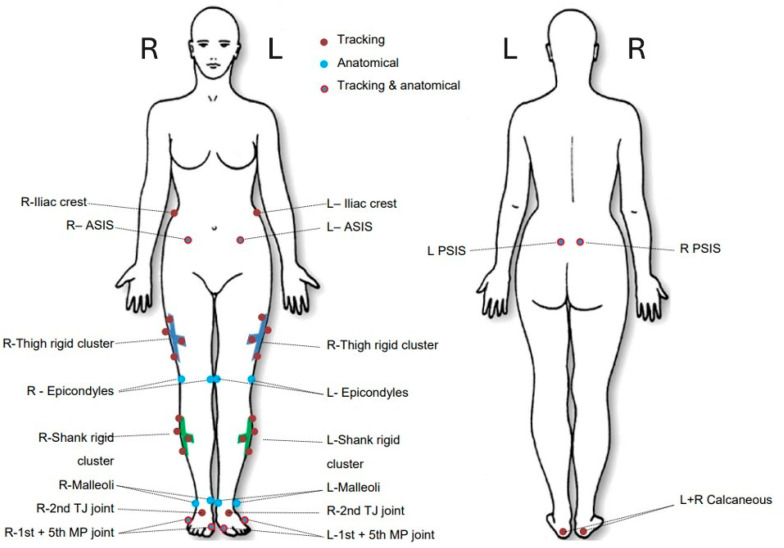
CAST marker set used during MoCap data collection [20,21].

Retro-reflective markers were placed on their lower bodies according to a CAST lower body marker set [20] (Figure 2), which is a standard marker set used in clinical gait analysis assessments.

**Figure 3:**
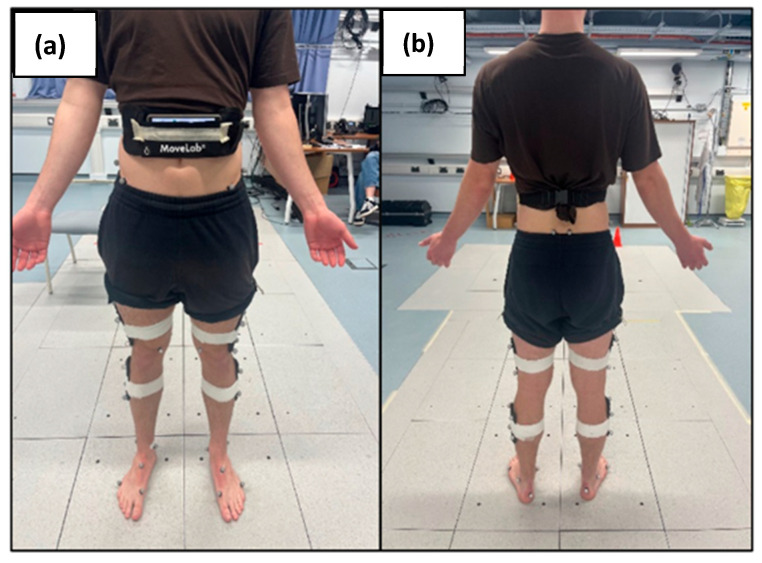
Static images of marker set and pouch placement with phone in-situ (**a**) anterior view (**b**) posterior view.

Each participant was also asked to ‘wear’ an android smartphone (Samsung Galaxy A25) provided by Agile Kinetic in a pouch provided and secured around the waist (Figure 3).

**Figure 4:**
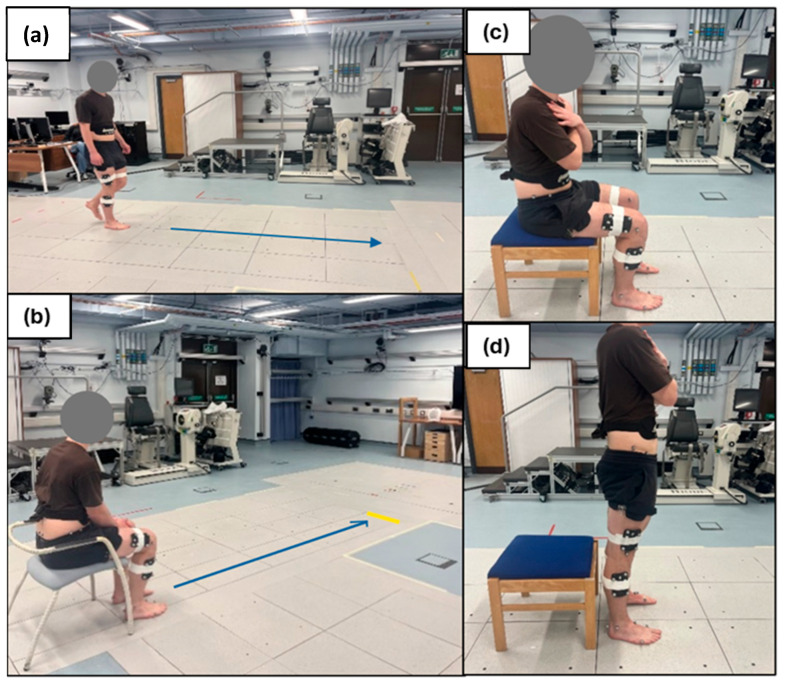
Walking trial (**a**), TUG (**b**), and STS (**c**,**d**).

Participants were asked to perform three sets of activities commonly used in clinical physiotherapy and rehabilitation settings to imitate movements characteristic of daily life: a thirty second walk (for gait parameter analysis), a Thirty-Second STS, and a TUG [16], shown in Figure 4.

The two methods simultaneously captured each activity and produced values across multiple metrics categorised as either STP, activity repetition count (for STS), or time taken to perform the activity (TUG).

Prior to performing each activity, participants accessed the DCA on the smartphone via a standard web browser. After entering their details and selecting the activity they were about to perform, participants pressed a key to start recording before placing the smartphone into the pouch. At the end of each activity, the smartphone was retrieved from the pouch and a key pressed to stop recording.

For the first activity, participants were asked to walk along the instrumented walkway at their natural pace and then continue walking within the laboratory for thirty seconds around a marked track. Gold Standard MoCap data was collected when the participant was walking across the instrumented walkway in the calibrated volume, while the MoveLab^®^ processing pipeline required a minimum of 30 s of data to generate gait parameters. For every participant, at least six walking trials were obtained (average = 8.72) until there were six clean force plate hits for left and right legs to ensure collection of valid kinetic data.

For the second activity, participants were asked to perform an STS assessment, timed for thirty seconds, starting in a seated position on a stool located on the force plates. A single trial was recorded using the Clinical Standard assessment method involving manual counting of the Sit-to-Stand repetitions.

The third activity, TUG, also starting from a seated position, involved standing up and walking forward for three meters and back to a seated position, with five separate trials recorded. The Clinical Standard assessment involved manual timing with a stopwatch.

The following STPs were produced in Visual3D: speed (m/s), stride length (m), step length (m), step time (s), cycle time (s), stance time (s), swing time (s), double support (s), initial double support (s), terminal double support (s), and cadence (100 steps/min). The STS repetition counts and TUG times were tabulated for the number of trials for each participant.

Recordings were excluded when either the MoCap system failed to capture the activity appropriately to allow processing or the DCA failed to upload sensor data to Firebase. For the latter case, this was due to the application occasionally failing to establish a stable internet connection.

The MoveLab^®^ DCA processing pipeline established for this study estimated gait STPs per subject, post-processing them into an accessible form to conduct comparison against the MoCap system. The estimated metrics summarise the trials per subject through a weighted average of all trials resulting in final gait STPs per subject. As part of the research and development process, nine different methods were adopted, labelled M1 to M9 in the results section, to estimate the gait STPs, STS, and TUG. The difference between them lies in the pre-processing algorithms, involving the rotation of inertial data through different planes using a ratio between height and leg length versus direct leg length measurement, along with different filtering and averaging techniques. Where methods involving the ratio between the height and leg length of the subjects were used, the outputs were considered in stages. Firstly, a blind comparison estimated gait metrics using a ratio derived in preliminary experiments. Then the ratio was refined to reflect the entire population of the 25 participating subjects.

The MoCap STPs were formatted for statistical analysis using MATLAB (MATLAB 2023. Version R2023a. The MathWorks Inc., Natick, MA, USA) to generate STP means and standard deviations.

To assess agreement between the methods, the normality of the results was tested prior to calculating Pearson correlation with statistical significance, indicated by a *p*-value less than 0.05 (Python Software Foundation, Wilmington, DE, USA. Python Language Reference, version 3.10. Available at https://www.python.org).

In addition, the Cronbach’s alpha was calculated across the set of MoveLab^®^ methods for each parameter to appraise the internal consistency and overall agreement between methods.

Based on the strength of the Pearson correlation between the MoveLab^®^ and Gold Standard MoCap STPs, the best-performing MoveLab^®^ method for each parameter was identified and the intraclass correlation coefficients (ICCs) were calculated with a *p*-value of 0.05 (SPSS version 29.0.2). This is intended to focus the reliability analysis of the MoveLab^®^ approaches that demonstrated good/close-to-good correlation with the Gold Standard MoCap outputs. The interpretation of the results is based on Koo and Li [22], with an ICC > 0.9 considered excellent, 0.75–0.9 good, 0.5–0.75 moderate, and <0.5 poor.

Bland–Altman plots were produced for the MoveLab^®^ approaches that demonstrated good/close-to-good correlation with the Gold Standard MoCap outputs (Python Software Foundation. Python Language Reference, version 3.10. Available at https://www.python.org), with reference to [18] to assess agreement between the two approaches by plotting the differences between the outputs from the two methods against their average for each parameter, where the Mean Difference (Bias), Limits of Agreement, and 95% Confidence Intervals were produced.

It should be noted the data collection protocol for STS dictated that the participant returned to sitting and manually stopped recording on the DCA when the laboratory team advised them that the 30 s had ended. For some trials, there was a misalignment between the person timing 30 s and the participant stopping the count. Thus, when the timer used by the laboratory stopped in the middle of a repetition, the DCA continued to record the final repetition. This resulted in the STS estimator reporting an extra count for these trials. This was acknowledged as a constraint of the experimental protocol involving the interaction with the DCA.

## 3. Results

Twenty-five healthy volunteers were recruited for the study with the following characteristic mean ± standard deviation: age = 31.8 ± 11.6 yrs, height = 1.73 ± 0.10 m, weight = 67.67 ± 12.70 kg, left leg length = 0.90 ± 0.06 m. MoveLab^®^ proposed nine slightly nuanced methods (M1 to M9), calculated using the range of algorithms tested in the MoveLab^®^ processing pipeline, to produce STP parameters for comparison with the Gold Standard MoCap.

Table 1 shows the mean difference between the MoveLab^®^ STP outputs when compared to the mean the MoCap outputs.

**Figure 5:**
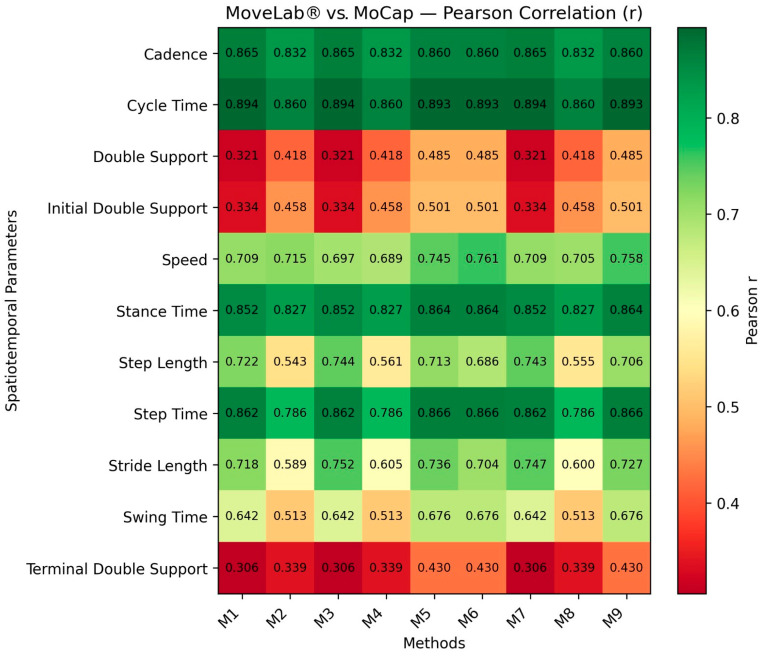
Heatmap of Pearson correlation r value of MoveLab^®^ methods against Gold Standard MoCap across all participants for each STP parameter. The colour bar represents a scale of [0.0, 1.0], where red propagates to green.

To evaluate the validity and reliability of the proposed methods, first the calculated Pearson correlation coefficient (r value) was calculated between each MoveLab^®^ method and the Gold Standard MoCap for all the STPs across all the participants. This is shown as a heatmap in Figure 5. All the correlations were found to be statistically significant (*p* < 0.05).

**Figure 6:**
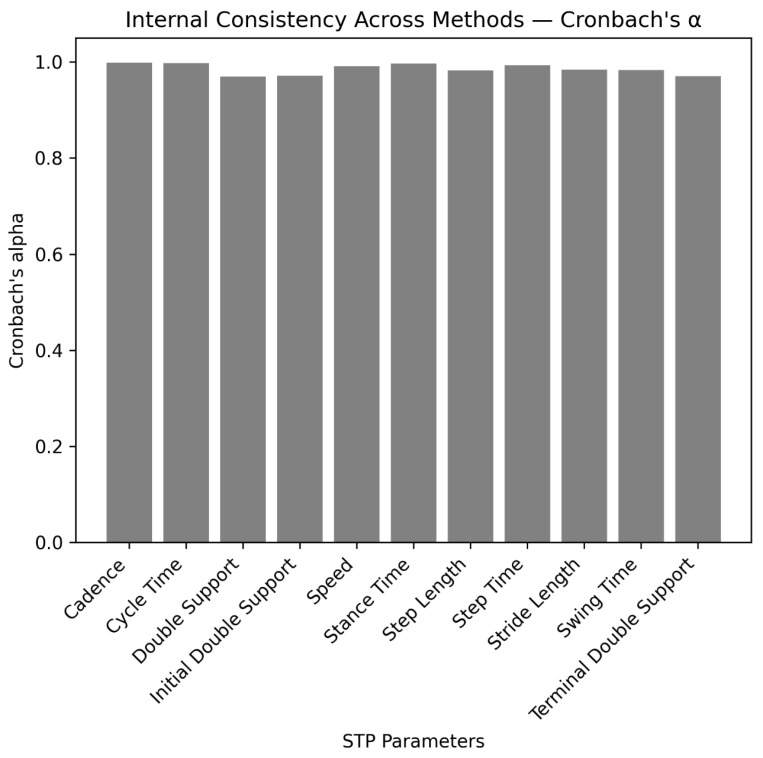
Bar chart of calculated Cronbach’s alpha demonstrating internal consistency of the different MoveLab^®^ methods.

In addition, the Cronbach’s alpha (Figure 6) is calculated across the set of MoveLab^®^ methods for each parameter to appraise the internal consistency and overall agreement between the methods.

Based on the strength of the Pearson correlation between the MoveLab^®^ and the Gold Standard MoCap STPs, the best-performing MoveLab^®^ method for each parameter was identified and the intraclass correlation coefficients (ICCs) were calculated (Table 2). This is intended to focus the reliability analysis of the MoveLab^®^ approaches that demonstrated a good/close-to-good correlation with the Gold Standard MoCap outputs. For all the methods, the spatial parameters showed a greater agreement with the Gold Standard compared to the temporal parameters. Moderate to good correlations (ICC = 0.590–0.894) are shown for eight parameters, including gait speed, stride length, stance time, cycle time, and cadence. A lower agreement is shown for the temporal outputs, double support time and initial and terminal double support times (ICC = 0.430–0.501), across all the methods.

**Figure 7:**
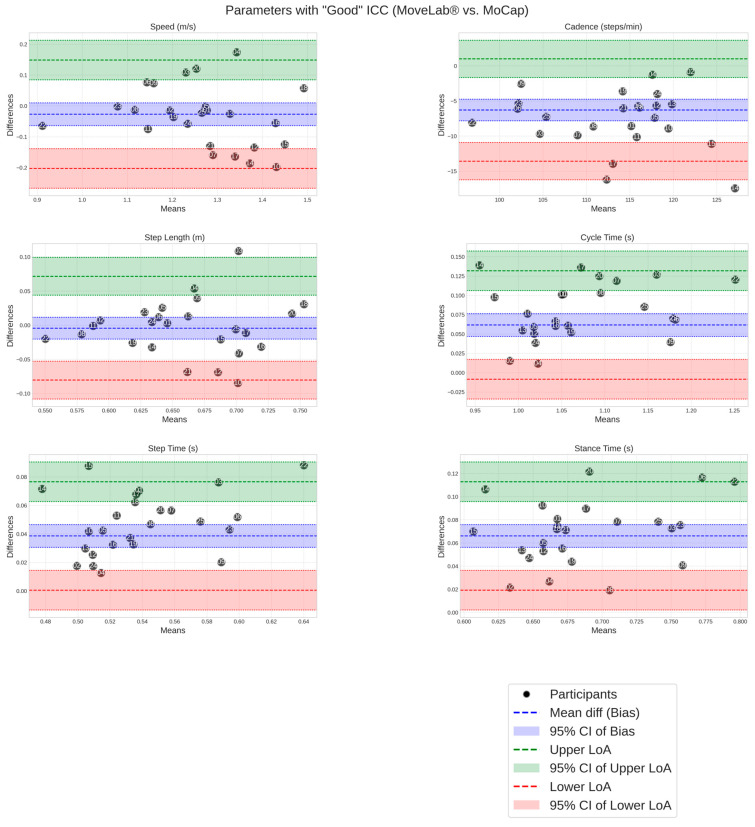
Bland–Altman plots show the spread of the MoveLab^®^ STPs, calculated using the method that produced the highest value for the good ICC, against Gold Standard MoCap across the 25 participants. The x-axis shows the mean parameter for each participant across both methods, while the y-axis shows the difference between the two methods. The long-dashed blue line represents the mean difference (bias), with dashed blue lines showing the limits of agreement (mean ± 1.96 SD). Coloured bands indicate different levels of agreement: red (small differences), blue (moderate), and green (larger).

Finally, for the best MoveLab^®^ methods that met the threshold for good (and near-good) correlation with the STP ICC, the Bland–Altman plots illustrate agreement with the Gold Standard MoCap and characterise the systematic bias and the limits of agreement (Figure 7).

**Figure 8:**
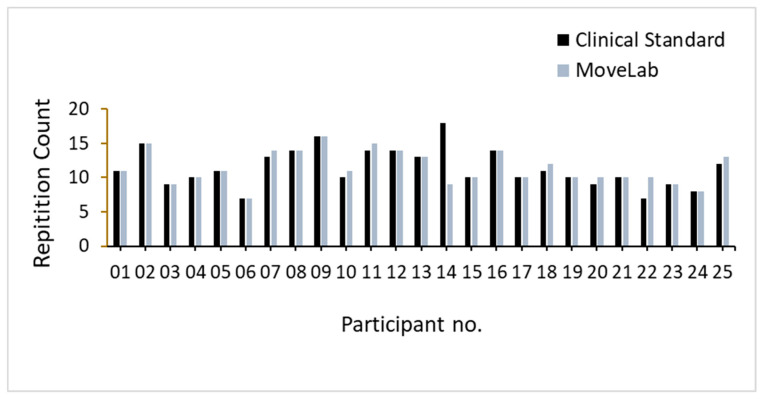
STS results recorded using both approaches, Clinical Standard assessment and MoveLab^®^, across all 25 participants.

The STS results across all the participants recorded using both approaches, the Clinical Standard assessment and MoveLab^®^, are shown in Figure 8. MoveLab^®^ demonstrated robustness in capturing the STS repetitions compared to the Clinical Standard assessment (ICC = 0.959). Excluding participant 14, the results indicate the same or a higher number of repetitions recorded over the 30 s (in seven participants), as compared to MoveLab^®^. Participant 14 shows almost double the number of repetitions recorded using the Clinical Standard compared to MoveLab^®^ and thus merits further examination.

**Figure 9:**
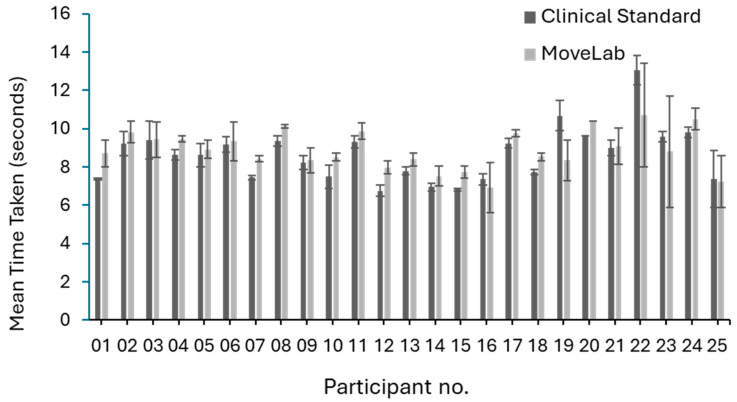
Mean TUG results with standard deviations recorded using both approaches, Clinical Standard assessment and MoveLab^®^, across all 25 participants for 5 repetitions.

The TUG results (mean and standard deviation) recorded using both of the approaches, the Clinical Standard assessment and MoveLab^®^, are presented in Figure 9. A similar trend is seen across most participants, with MoveLab^®^ estimating shorter times compared to the Clinical Standard results, with exception of participants 19, 22, 23, and 25. Given the variability in the time taken to complete the TUG observed in the comparative bar chart, it reflects the range expected for a healthy cohort [23] (average age of 31.8 years), which is <12 s. An ICC of 0.757 across the 25 participants indicated a good agreement was presented by MoveLab^®^ when compared to the Clinical Standard.

**Figure 10:**
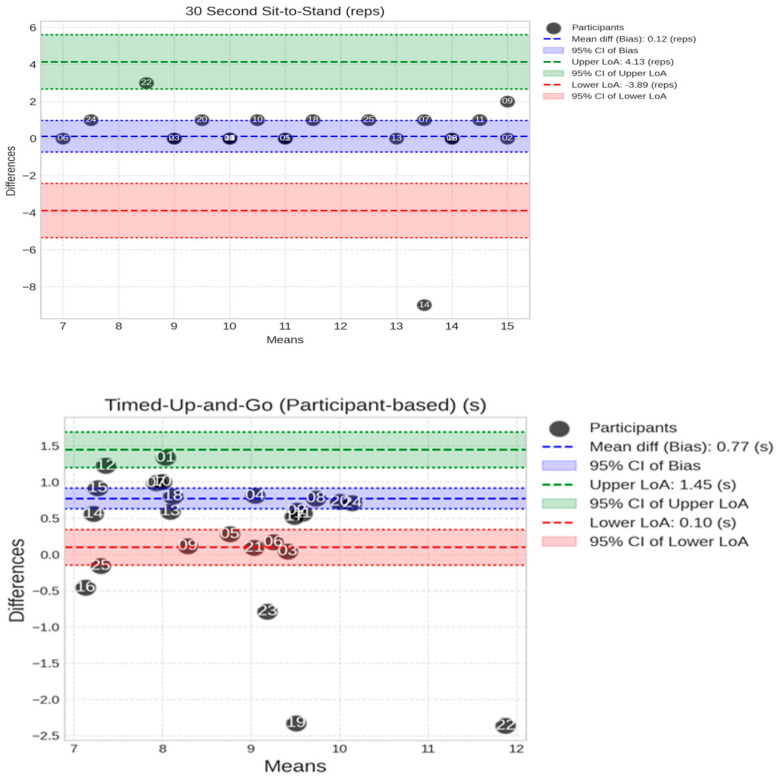
Bland–Altman plots show the spread of MoveLab^®^ data outputs for STS (repetition count) (**top plot**) and TUG (s) (**bottom plot**) against Clinical Standard assessment outputs for all participants. The plots compare cycle time measurements between two methods across the 25 participants. The x-axis shows the mean output for each participant across both methods, while the y-axis shows the difference between the two methods. The long-dashed blue line represents the mean difference (bias), with dashed blue lines showing the limits of agreement (mean ± 1.96 SD). Coloured bands indicate different levels of agreement: red (small differences), blue (moderate), and green (larger).

The Bland–Altman plots, shown in Figure 10, indicate the spread of the difference between the MoveLab^®^ data outputs for the STS and the TUG against the Clinical Standard assessment for all the participants. Moderate differences are seen for the STS. Small to moderate differences can be observed for the TUG.

Supplementary graphics including Bland-Altman plots for all metrics, Pearson correlation and Cronbach’s Alpha are presented in the Appendix A.

## 4. Discussion

The aim of this study was to assess the reliability and validity of the MoveLab^®^ (Agile Kinetic 2024) approach to measure gait spatiotemporal parameters (STPs), STS, and TUG using a waist-worn mobile phone, compared to the Gold Standard 3D marker-based motion capture (Qualisys AB, Sweden) and the Clinical Standard assessment methods. The MoveLab^®^ DCA processing pipeline established for this study estimated gait STPs per subject and was trialed using nine different methods to estimate the gait STPs, the TUG, and the STS, and then refined to reflect the entire population of the 25 participating subjects.

The ability to accurately measure STPs outside of a laboratory or clinical environment offers significant advantages in accessibility, cost-effectiveness, and efficiency in diagnosing and monitoring treatment outcomes [2]. The sensor-based gait analysis system developed by MoveLab^®^ presents a promising solution for real-world, low cost, and unsupervised gait assessments, potentially enabling the detection of mobility impairments and aiding in rehabilitation strategies and monitoring.

Few studies have directly compared smartphone motion sensors with the Gold Standard Clinical MoCap (Clinical Gait Analysis) or the Clinical Standard assessments such as the TUG and the STS. A systematic review of IMU-based systems [24] reported a high validity relative to 3D MoCap for slow, sagittal-plane movements but a reduced accuracy for dynamic, multi-plane tasks.

The OpenCap device has been reported for the measurement of human movement dynamics from smartphone videos [25] using pose estimation, deep learning, and biomechanical models to estimate 3D kinematics, muscle activations, joint loads, and moments. OpenCap allows synchronous video collection from two or more smartphones and has potential for screening disease risk, evaluating interventions, and supporting rehabilitation decisions. Competitive athletes performing jump-land-jump tasks showed that IMU- and phone-based systems generated sagittal-plane joint kinematic waveforms comparable in shape to the optical MoCap, though the hip flexion magnitudes differed and the transverse/frontal plane validity was limited [26].

The smartphone application (OneStep, Celloscope, Israel) has been validated using two thigh-mounted smartphones, showing good-to-excellent ICCs for spatiotemporal gait measures compared with the marker-based MoCap, supporting longitudinal clinical monitoring [27]. Single smartphone studies using a front-pocket placement also showed reasonable validity against multi-sensor IMUs [7], demonstrating their usability in natural settings, though the absolute agreement can vary, and the findings are limited to controlled lab conditions. The results of the current study suggested that further validation against the marker-based MoCap is needed.

The novelty of MoveLab^®^ is that it offers a quick and practical assessment of mobility using accessible Web browsers on any platform or on the user’s smart mobile phone, to assist with the remote, longitudinal monitoring of patient status, and thus it allows timely intervention. Smartphone- and IMU-based systems have a strong potential for sagittal-plane and spatiotemporal gait assessment, but their accuracy is limited for diagnostics; their greatest value is in longitudinal individual monitoring, where relative changes over time are the most informative. To our knowledge, the present study is the first to report a comparison of the MoveLab^®^ platform using a single smartphone IMU positioned on the trunk against the Gold Standard marker-based MoCap along with the Clinical Standard assessments (TUG and STS).

In this study, MoveLab^®^ demonstrated moderate-to-good correlations with the Gold Standard MoCap measurements for nine STP parameters, including gait speed, stride length, stance time, and cadence. These parameters are critical for assessing locomotor impairments in conditions such as cerebral palsy, stroke, and Parkinson’s disease, where subtle gait alterations can serve as early indicators of disease progression [4]. The accurate, repeatable measurement of these gait characteristics in non-clinical settings, at an appropriate threshold of accuracy compared to the Gold Standard, could support continuous patient monitoring and remote rehabilitation, improving accessibility for individuals with mobility disorders.

Although the MoveLab^®^ DCA processing pipeline established for this study showed good results for several of the key gait parameters, the algorithms exhibited poor correlations for double support and terminal double support phases, with a moderate correlation (0.501) for initial double support. These discrepancies could be attributed to the individual participants’ anatomical and functional variations. For example, left–right asymmetry, or differences in step timing detection, are particularly relevant in pathologies characterised by asymmetric gait patterns, such as hemiplegic stroke and unilateral orthopedic conditions [28]. The lower accuracy of the MoveLab^®^ algorithm in these phases suggests that while MoveLab^®^ may be effective for general gait assessments, the DCA was positioned at the participant’s waist; therefore, parameters that depend on side-specific measurements were estimated as general averages of gait rather than as leg-specific metrics. It is important to note that MoveLab^®^ is currently designed for remote use at the patient’s convenience, making it well suited to conditions that do not require in-person clinical assessments. By contrast, disorders such as Parkinson’s disease, hemiparetic gait, and age-related syndromes, where metrics such as double support time and step span are critical for evaluating imbalance [29,30,31,32] necessitate a direct clinical evaluation, and in such cases MoveLab^®^ may not currently be the most appropriate tool for the mobility analysis. The ongoing algorithm development aims to enable the estimation of the leg-specific parameters, facilitated by the device placement in the patient’s pocket.

The STS and TUG are two of the OARSI [23] recommended set of performance-based tests of physical function that are best suited for older individuals (>40 years) diagnosed with hip and/or knee osteoarthritis (OA), including end stage disease or following joint replacement. They are intended for use by both clinicians and researchers as performance outcome measures and are viewed as complementary to established self-report measures such as questionnaires.

The STS test—counting the number of times that a person can repeatedly stand from being seated on a chair and then sitting down again over a 30 s period—is used to assess lower body strength and is part of the Short Physical Performance Battery (SPPB), commonly used for the assessment of physical performance in older adults. The MoveLab^®^ processing pipeline for the Thirty-Second STS was validated against the 25 participant trials, i.e., one trial per subject. The impact of the data variability across the participant cohort [23] was evaluated to provide a fair and accurate assessment of the approach to using the MoveLab^®^ platform with the data collected via the waist-worn mobile phone. The output for subject 14 appears to be significantly worse compared with the other 24 subjects, where the system demonstrated a comparable performance to the Gold Standard. Upon investigation, the signal-to-noise ratio of this particular trial was found to be considerably lower compared to that for the other participants. This suggests that an external factor may have contributed to the noise for this recording. Due to the difficulty in recalling the subject for a retrial, it was decided to present the analysis including participant 14 data in the comparative variability observations (Figure 7) but omitting it from the overall ICC calculations. Given the small sample size, the influence of individual trials on the analysis is not trivial. While MoveLab^®^ demonstrated robustness in capturing the STS repetitions compared to the Clinical Standard assessment (ICC = 0.959), the ability to process outliers impacted by environmental conditions and trial discrepancies appears to be poor, as evidenced by participant 14.

The TUG test—a simple assessment often used to screen for frailty and fall risk in older adults—involves measuring the time it takes to rise from a chair, walk three meters, turn, walk back, and sit down. A longer TUG time, generally > 12–14 s, is associated with increased frailty and fall risk. The observed mean TUG results are variable and lie within the range expected for a healthy cohort when recorded using both the MoCap and MoveLab^®^ approaches (less than 12). The ICC (0.757) across the 25 participants, aligns with the small-to-moderate differences observed in the Bland–Altman plot, and it demonstrates good agreement, indicating that the MoveLab^®^ data processing pipeline is capable of providing a valid approach to measuring the TUG.

The misalignment of repetition counts observed for the STS when using the DCA developed for this study can be mitigated in the future commercial MoveLab^®^ application through a built-in timer that alerts the user when the activity is starting and a countdown timer which automatically stops after 30 s, providing a second alert. It is intended that the commercial application will include a two-stage methodology for handling the outliers impacted by environmental conditions and trial discrepancies in STS assessments. If a first stage involving signal filtering does not adequately suppress the noise, the user will be asked to perform the assessment again. Where internet connectivity issues are present, these will be overcome automatically through a retry mechanism, whereby, if the sensor data fails to upload, the application will attempt to re-upload.

The limitations of this study should be considered and can present opportunities for future research to build on the current findings.

Firstly, the difference in the capture time between the MoveLab^®^ device and the Gold Standard and the Clinical Standard assessment methods may have contributed to the differences in output agreement, thus impacting the strength of correlations. The capture period for the MoveLab^®^ device included some turns in the walking route and this has already been addressed in the MoveLab^®^ algorithms. However, acceleration and changes in speed and gait pattern could affect the STP outputs.

Secondly, the recognised limitations of the 3D gait analysis methods could contribute to errors in the MoCap data, including a soft tissue artifact and incorrect or inaccurate marker placement. However, for the STP outputs in this study, the impact would be expected to be minimal when compared to the joint rotations that are analyzed in a full clinical gait analysis. Future comparative studies could involve comparison of MoveLab^®^ with portable gait analysis devices or wearable motion analysis systems however they are not generally considered as Gold Standard for clinical assessment.

A further limitation may be considered in relation to the STS assessment, where a single trial was recorded. It is accepted that multiple trials allow for individual variation when performing a task; however, due to the battery of assessments involving gait, the TUG, and the STS, it was considered sufficient for this assessment and removed any effect of fatigue.

Finally, this study was limited to a cohort of twenty-five volunteers who were recruited as self-reported healthy participants. To address the potential limitations that may arise with the current algorithms and processing pipeline when applied to cohorts across typical pathologies, e.g., osteoarthritis, stroke, and Parkinson’s, it is also recommended to perform further developmental and comparative studies. These should involve altered or compensatory gait styles, for example, with simulated or real gait disorders, and clearly identified clinical patient cohorts (for clinical benchmarking to the current markers and assessments). This should also include an assessment of alternative options for mobile phone placement to allow for a range of patient morphologies, abilities, and clothing.

In identifying these key limitations, it must be noted that the data collection protocol and processing pipeline was adopted to assess the ability of MoveLab^®^ to recreate a set of reliable and clinically valid outputs when compared to the Gold Standard MoCap and the Clinical Standard assessments. Although we must interpret the findings of the present study with caution, the resulting data has provided the first evidence of validation with good correlations for several key gait STPs and two clinically accepted performance-based assessments, along with clearly defined opportunities to address the identified limitations.

## 5. Conclusions

This study provides the first evidence that, in healthy participants, the MoveLab^®^ pipeline yields acceptable-to-good accuracy for spatiotemporal gait parameters (STPs) compared with gold-standard motion capture and demonstrates excellent and good performance for the Sit-to-Stand (STS) and the Timed-Up-and-Go (TUG), respectively. These results are encouraging but should be interpreted with caution, as the methods cannot yet be directly generalized to clinical cohorts. Our ongoing work is validating the pipeline against both the Gold Standard MoCap and GAITRite^®^ and examining the influence of phone placement (pocket and waist locations), as well as studying the sensitivity to the induced gait alterations and the compensatory gait styles representative of those observed in patient groups who may ultimately benefit from home monitoring.

Based on the findings and recommendations of this initial comparative study, the DCA and the processing pipeline can be considered a preliminary step towards establishing reliable measurement of STPs in healthy adults performing activities within controlled environments. However, further validation is required before its use can be extended to clinical populations or less constrained settings. Importantly, this approach highlights the potential to develop practical, rapid, and scalable methods for data collection and analysis that could, with sufficient validation, support clinicians and researchers in monitoring the activities of daily living beyond the laboratory or clinic, and in the longer term, enable the remote assessment of patient groups where compensatory gait adaptations are clinically significant.

## Figures and Tables

**Table 1:** The mean differences between the MoveLab^®^ STP outputs when compared to the MoCap means, where M represents the utilised algorithm approach (highest mean differences shaded grey).

**STP**	**MoCap** **Mean** **± SD**	**Mean Differences Between the MoveLab^®^ STP Outputs when Compared to the MoCap Means**								
		M1	M2	M3	M4	M5	M6	M7	M8	M9
**Speed (m/s)**	1.28 ± 0.11	+0.084	−0.033	+0.095	−0.027	+0.048	+0.035	+0.102	−0.020	+0.041
**Stride Length (m)**	1.31 ± 0.03	−0.019	−0.140	−0.005	−0.140	−0.053	−0.067	+0.005	−0.130	−0.060
**Step Length (m)**	0.66 ± 0.02	−0.003	−0.068	+0.004	−0.064	−0.245	−0.031	+0.007	−0.064	−0.028
**Step Time (s)**	0.52 ± 0.02	−0.050	−0.049	−0.050	−0.049	−0.048	−0.048	−0.049	−0.049	−0.048
**Cycle Time (s)**	1.03 ± 0.02	−0.080	−0.078	−0.080	−0.078	−0.077	−0.077	−0.079	−0.077	−0.077
**Stance Time (s)**	0.65 ± 0.02	−0.046	−0.093	−0.046	−0.093	−0.069	−0.069	−0.046	−0.094	−0.070
**Swing Time (s)**	0.38 ± 0.01	−0.038	+0.009	−0.038	+0.009	−0.012	−0.012	−0.037	+0.010	−0.012
**Double Support (s)**	0.27 ± 0.02	−0.024	−0.117	−0.024	−0.117	−0.070	−0.070	−0.025	−0.118	−0.071
**Initial Double Support (s)**	0.14 ± 0.01	−0.012	−0.057	−0.012	−0.057	−0.035	−0.035	−0.012	−0.058	−0.035
**Terminal Double Support (s)**	0.13 ± 0.01	−0.013	−0.059	−0.013	−0.059	−0.026	−0.036	−0.013	−0.058	−0.036
**Cadence (100 steps/min)**	1.17 ± 0.04	+0.076	+0.072	+0.076	+0.072	+0.073	+0.073	+0.076	+0.071	+0.073

**Table 2:** Intraclass Correlation Coefficient calculated for the MoveLab^®^ methods; those that produced the highest Pearson correlation coefficient are shaded in green.

STP	Best Method	ICC
Speed (m/s)	M6	0.761
Stride Length (m)	M3	0.590
Step Length (m)	M3	0.745
Step Time (s)	M5	0.866
Cycle Time (s)	M7	0.894
Stance Time (s)	M5	0.864
Swing Time (s)	M5	0.676
Double Support (s)	M9	0.485
Initial Double Support (s)	M9	0.501
Terminal Double Support (s)	M5	0.430
Cadence (100 steps/min)	M3	0.859

**Figure A1:**
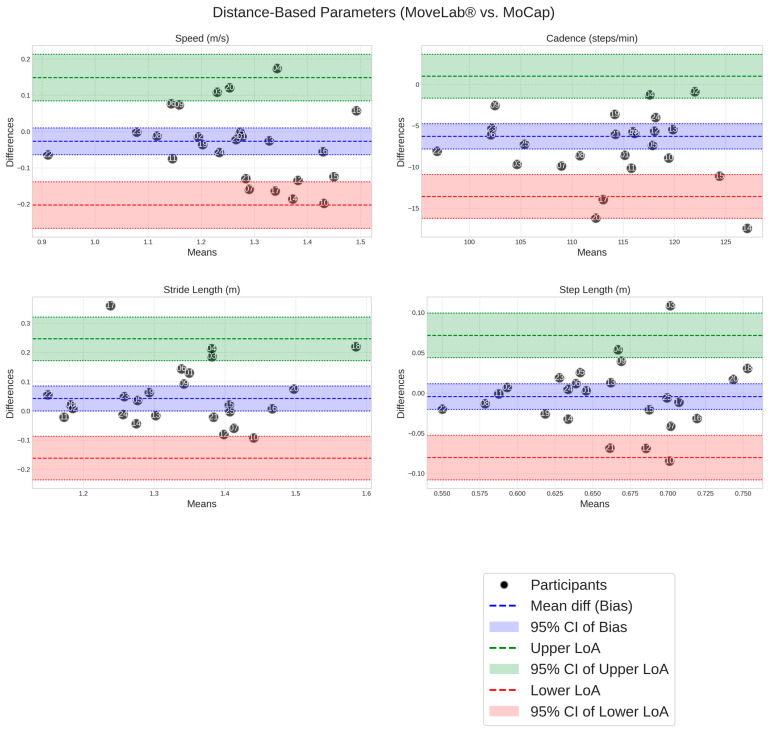
Bland–Altman plots to show the spread of MoveLab^®^ gait spatial parameter outputs against Gold Standard MoCap for all participants.

**Figure A2:**
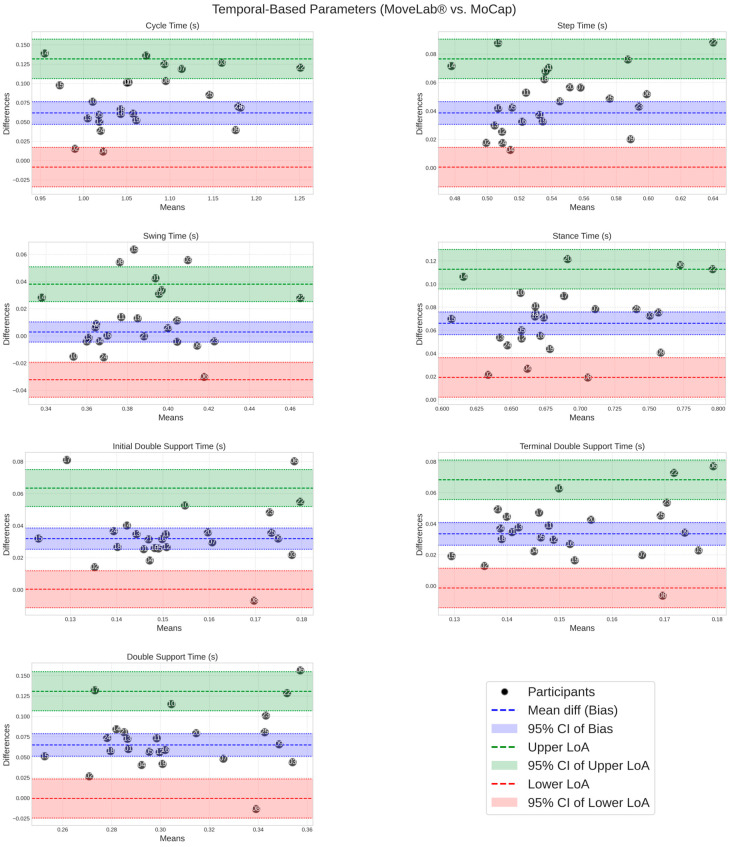
Bland–Altman plots to show the spread of MoveLab^®^ gait temporal parameter puts against Gold Standard MoCap for all participants.

**Figure A3:**
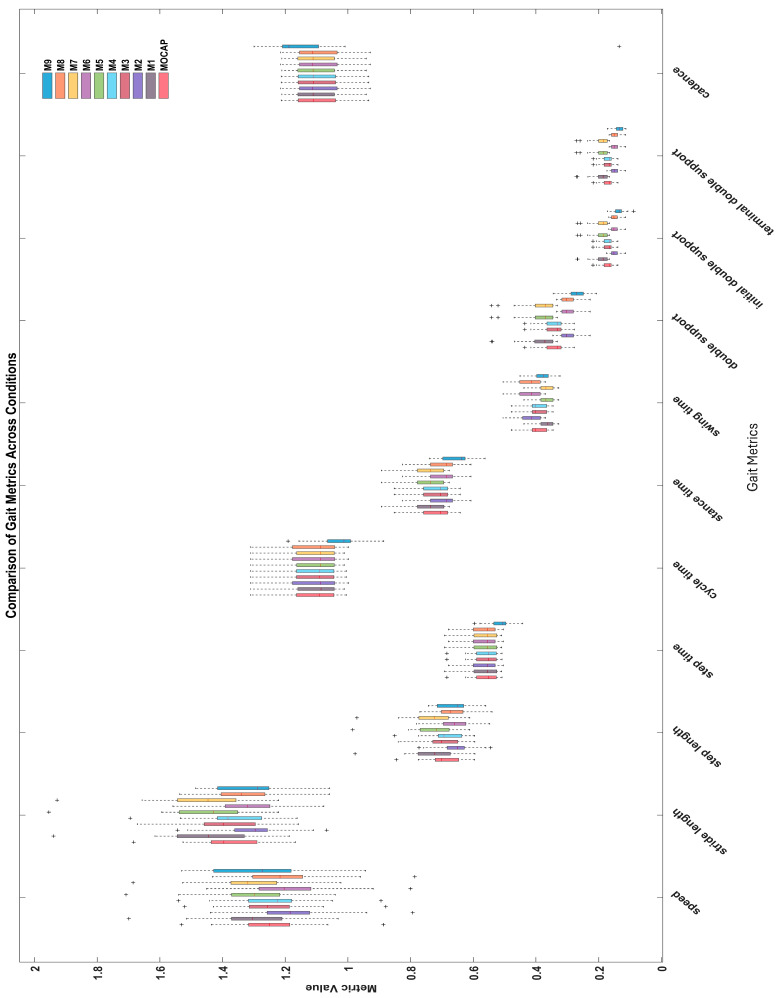
Boxplot of gait STPs from Gold Standard MoCap and MoveLab^®^ demonstrator algorithms for all participants. The red line marks the median for each gait metric, and the coloured box represents the interquartile range for each gait metric. The dashed lines represent the range of the remaining data and the crosses represent outliers.

**Figure A4:**
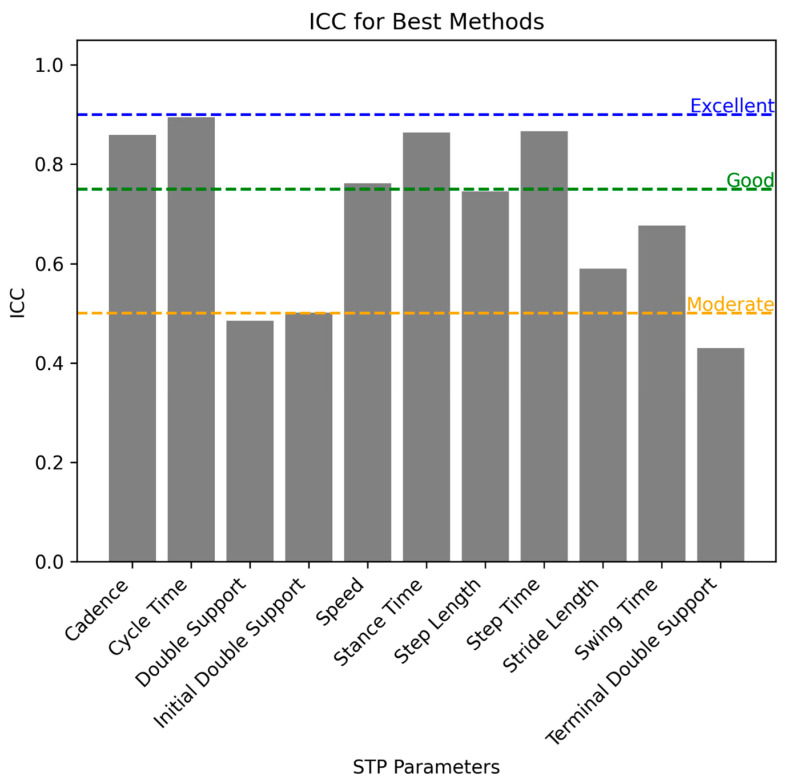
Bar chart of computed ICC values for the selected best method against each STP parameter. The dashed lines represent the agreed standard range of moderate, good, and excellent agreement.

## Data Availability

The datasets used and analyzed during the current study are available from the corresponding author on reasonable request.

## Appendix A

This provides supplementary graphical information including Bland–Altman plots showing spread of MoveLab^®^ STP data against Gold Standard MoCap for all participants (Figure A1 and Figure A2), a Boxplot of gait STPs for Gold Standard MoCap and MoveLab^®^ demonstrator algorithms for all participants (Figure A3), Pearson correlation calculations of MoveLab^®^ against Gold Standard MoCap STP parameters for all participants (Table A1 and Table A2), the ICC calculated for each outperforming method against each STP parameter (Figure A4), and Cronbach’s alpha that demonstrates MoveLab^®^’s method consistency (Table A3).

**Table A1:** Pearson correlation r value of MoveLab^®^ methods against MoCap for each STP parameter.

STP	Pearson r Value								
	M1	M2	M3	M4	M5	M6	M7	M8	M9
Speed (m/s)	0.709	0.715	0.697	0.689	0.745	0.761	0.709	0.705	0.758
Stride Length (m)	0.718	0.589	0.752	0.605	0.736	0.704	0.747	0.600	0.727
Step Length (m)	0.722	0.543	0.744	0.561	0.713	0.686	0.743	0.555	0.706
Step Time (s)	0.862	0.786	0.862	0.786	0.866	0.866	0.862	0.786	0.866
Cycle Time (s)	0.894	0.860	0.894	0.860	0.893	0.893	0.894	0.860	0.893
Stance Time (s)	0.852	0.827	0.852	0.827	0.864	0.864	0.852	0.827	0.864
Swing Time (s)	0.642	0.513	0.642	0.513	0.676	0.676	0.642	0.513	0.676
Double Support (s)	0.321	0.418	0.321	0.418	0.485	0.485	0.321	0.418	0.485
Initial Double Support (s)	0.334	0.458	0.334	0.458	0.501	0.501	0.334	0.458	0.501
Terminal Double Support (s)	0.306	0.339	0.306	0.339	0.430	0.430	0.306	0.339	0.430
Cadence (100 steps/min)	0.865	0.832	0.865	0.832	0.860	0.860	0.865	0.832	0.860

**Table A2:** Pearson correlation *p*-value of MoveLab^®^ methods against MoCap for each STP parameter.

STP	Pearson’s *p*-Value								
	M1	M2	M3	M4	M5	M6	M7	M8	M9
Speed (m/s)	7 × 10^−5^	6 × 10^−5^	1 × 10^−4^	1 × 10^−4^	2 × 10^−5^	1 × 10^−5^	7 × 10^−5^	8 × 10^−5^	1 × 10^−5^
Stride Length (m)	5 × 10^−5^	2 × 10^−3^	1 × 10^−5^	1 × 10^−3^	3 × 10^−5^	9 × 10^−5^	2 × 10^−5^	2 × 10^−3^	4 × 10^−5^
Step Length (m)	5 × 10^−5^	5 × 10^−3^	2 × 10^−5^	4 × 10^−3^	6 × 10^−5^	2 × 10^−4^	2 × 10^−5^	4 × 10^−3^	8 × 10^−5^
Step Time (s)	3 × 10^−8^	3 × 10^−6^	3 × 10^−8^	3 × 10^−6^	2 × 10^−8^	2 × 10^−8^	3 × 10^−8^	3 × 10^−6^	2 × 10^−8^
Cycle Time (s)	2 × 10^−9^	4 × 10^−8^	2 × 10^−9^	4 × 10^−8^	2 × 10^−9^	2 × 10^−9^	2 × 10^−9^	4 × 10^−8^	2 × 10^−9^
Stance Time (s)	7 × 10^−8^	3 × 10^−7^	7 × 10^−8^	3 × 10^−7^	3 × 10^−8^	3 × 10^−8^	7 × 10^−8^	3 × 10^−7^	3 × 10^−8^
Swing Time (s)	5 × 10^−4^	9 × 10^−3^	5 × 10^−4^	9 × 10^−3^	2 × 10^−4^	2 × 10^−4^	5 × 10^−4^	9 × 10^−3^	2 × 10^−4^
Double Support (s)	1 × 10^−1^	4 × 10^−2^	1 × 10^−1^	4 × 10^−2^	1 × 10^−2^	1 × 10^−2^	1 × 10^−1^	4 × 10^−2^	1 × 10^−2^
Initial Double Support (s)	1 × 10^−1^	2 × 10^−2^	1 × 10^−1^	2 × 10^−2^	1 × 10^−2^	1 × 10^−2^	1 × 10^−1^	2 × 10^−2^	1 × 10^−2^
Terminal Double Support (s)	1 × 10^−1^	1 × 10^−1^	1 × 10^−1^	1 × 10^−1^	3 × 10^−2^	3 × 10^−2^	1 × 10^−1^	1 × 10^−1^	3 × 10^−2^
Cadence (100 steps/min)	3 × 10^−8^	3 × 10^−7^	3 × 10^−8^	3 × 10^−7^	3 × 10^−8^	3 × 10^−8^	3 × 10^−8^	3 × 10^−7^	3 × 10^−8^

**Table A3:** Cronbach’s alpha value of MoveLab^®^ methods consistency for each STP parameter.

STP	Cronbach Alpha
Speed (m/s)	0.991
Stride Length (m)	0.984
Step Length (m)	0.982
Step Time (s)	0.993
Cycle Time (s)	0.998
Stance Time (s)	0.996
Swing Time (s)	0.983
Double Support (s)	0.970
Initial Double Support (s)	0.971
Terminal Double Support (s)	0.970
Cadence (100 steps/min)	0.998
